# Fluid overload and survival in critically ill patients with acute kidney injury receiving continuous renal replacement therapy

**DOI:** 10.1371/journal.pone.0172137

**Published:** 2017-02-14

**Authors:** Il Young Kim, Joo Hui Kim, Dong Won Lee, Soo Bong Lee, Harin Rhee, Eun Young Seong, Ihm Soo Kwak, Sang Heon Song

**Affiliations:** 1 Department of Internal Medicine, Pusan National University School of Medicine, Yangsan, Republic of Korea; 2 Research Institute for Convergence of Biomedical Science and Technology, Pusan National University Yangsan Hospital, Yangsan, Republic of Korea; 3 Medical Research Institute, Pusan National University Hospital, Busan, Republic of Korea; University of Sao Paulo Medical School, BRAZIL

## Abstract

**Background:**

Fluid overload is known to be associated with increased mortality in patients with acute kidney injury (AKI) who are critically ill. In this study, we intended to uncover whether the adverse effect of fluid overload on survival could be applied to all of the patients with AKI who received continuous renal replacement therapy (CRRT).

**Methods:**

We analyzed 341 patients with AKI who received CRRT in our intensive care units. The presence of fluid overload was defined as a minimum 10% increase in body weight from the baseline. Demographics, comorbid diseases, clinical data, severity of illness [the sequential organ failure assessment (SOFA) score, number of vasopressors, diagnosis of sepsis, use of ventilator] upon ICU admission, fluid overload status, and time elapsed from AKI diagnosis until CRRT initiation were reviewed from the medical charts.

**Results:**

Patients with total fluid overload from 3 days before CRRT initiation to ICU discharge had a significantly lower survival rate after ICU admission, as compared to patients with no fluid overload (*P* < 0.001). Among patients with sepsis (*P* < 0.001) or with high SOFA scores (*P* < 0.001), there was a significant difference in survival of the patients with and without fluid overload. In patients without sepsis or with low SOFA score, there was no significant difference in survival of patients irrespective of fluid overload.

**Conclusion:**

Our study demonstrates that the adverse effect of fluid overload on survival is more evident in patients with sepsis or with more severe illness, and that it might not apply to patients without sepsis or with less severe illness.

## Introduction

Acute kidney injury (AKI) is frequently encountered in patients who are critically ill [1, 2]. Despite significant advances in intensive care medicine, the prognosis of AKI remains poor, with significant mortality and morbidity [3]. Approximately 4% of critically ill patients with AKI have been shown to require renal replacement therapy and frequently receive continuous renal replacement therapy (CRRT) due to the principal advantage of its hemodynamic stability [4, 5].

Although maintaining an appropriate fluid balance is a mainstay for the management of critically ill patients with AKI, there is no consensus on optimal fluid management. The traditional approach has involved aggressive fluid resuscitation aimed at maintaining renal perfusion and preventing further ischemic injury during ongoing renal dysfunction [6, 7]. However, recent studies have demonstrated an association between fluid overload and mortality in critically ill children and adults with AKI [8–12]. Thus, more strict and timely fluid management in such patients is recommended to avoid fluid overload.

In this study, we aimed to evaluate the association between fluid overload and mortality in critically ill patients with AKI receiving CRRT. The hypotheses of this study were as follows: First, fluid overload may be associated with mortality in AKI patients receiving CRRT. Second, the proper fluid removal by CRRT may reduce mortality in such patients. Third, the adverse effect of fluid overload on survival may be more evident in some subgroups of patients with AKI, such as those with sepsis or a greater severity of illness.

## Material and methods

### Study population and data collection

We retrospectively reviewed all adult patients with AKI (age: 18–92 years) who received CRRT in our medical or surgical intensive care unit (ICU) from January 2007 to December 2013 at Pusan National University Hospital and Pusan National University Yangsan Hospital. Patients with pre-existing end-stage renal disease were excluded. The primary indications for CRRT were severe uremia, severe fluid overload, electrolyte imbalance, or metabolic acidosis that was refractory to conservative management. This study protocol was approved by the hospital’s institutional review board (Pusan National University Yangsan Hospital Review Board, IRB No. 05-2015-013). Informed consent was waived by the IRB due to the retrospective nature of the analysis by using information contained in medical charts and records, which were anonymized.

All patients were treated with Gambro PRISMA dialysis machines using AN-69 polyacrylonitrile membrane dialyzer. The CRRT modalities were continuous veno-veno hemodialysis, continuous veno-veno hemofiltration, and continuous hemodiafiltration. Decisions regarding when to initiate or terminate CRRT, the modality, and the prescribed dose of CRRT were made on an individual basis by the attending nephrologist. The CRRT dose of 25–30 ml/kg per hour was prescribed to ensure a delivered CRRT dose of ≥ 20 ml/kg per hour.

Demographics, comorbid diseases, ICU location (medical ICU or surgical ICU), clinical data, severity of illness [the sequential organ failure assessment (SOFA) score, number of vasopressors, diagnosis of sepsis, and use of a ventilator] at ICU admission, fluid overload status, and time elapsed from AKI diagnosis until the initiation of CRRT were obtained from the medical charts.

### Definitions and outcomes

AKI was defined according to the Kidney Disease: Improving Global Outcome (KDIGO) guidelines (increase in serum creatinine ≥ 0.3 mg/dl within 48 hours; or increase in serum creatinine ≥ 1.5 times baseline; or urine volume < 0.5 ml/kg/h for 6 hours) [13]. All available input and output data, starting from 3 days before CRRT initiation to ICU discharge, were analyzed in this study. Foley catheters were inserted in all patients for urine output monitoring. The degree of fluid overload was expressed as percent fluid overload (%FO), which was calculated by the following formula: [∑ daily (fluid intake (L)-total output (L))/baseline body weight (in kilograms)] x 100. Baseline body weight was obtained from the first available documented weight after visiting the hospital. The presence of fluid overload was defined as %FO ≥ 10% over the baseline body weight. This cutoff point was derived from the previous Program to Improve Care in Acute Renal Disease (PICARD) study [12]. The %FO was further subdivided into percent fluid overload during 3 days prior to CRRT initiation (%FOpreCRRT), and percent fluid overload from CRRT initiation to ICU discharge (%FOpostCRRT). Finally, total fluid overload from 3 days before CRRT initiation to ICU discharge (%FOtotal) was defined as %FOpreCRRT+%FOpostCRRT.

For the subgroup analysis, all subjects were classified into 4 groups: Group 1 (%FOpreCRRT < 10% and %FOtotal < 10%; no fluid overload before and after CRRT application, and finally, no total fluid overload); Group 2 (%FOpreCRRT ≥ 10% and %FOtotal < 10%; fluid overload before CRRT that was then resolved by CRRT, and finally, no total fluid overload); Group 3 (%FOpreCRRT < 10% and %FOtotal ≥ 10%; no fluid overload before CRRT, but total fluid overload due to aggravation of fluid overload during CRRT); and Group 4 (%FOpreCRRT ≥ 10% and %FOtotal ≥ 10%; fluid overload before CRRT, which was not resolved by CRRT, and finally, total fluid overload). We also classified patients into high SOFA score (≥ 13) and low SOFA score (< 13) groups using the median SOFA score.

### Statistical analysis

Continuous variables were expressed as mean ± SD and compared using Student’s t test to determine whether differences existed between survivors and non-survivors. Categorical variables were expressed as a proportion (%) and were compared using the chi-square test. We performed a Kaplan-Meier survival analysis within 30 days of ICU admission and compared the survival curve between patients with or without fluid overload using the Log rank test. To determine the independent risk factors for 30-day mortality after ICU admission, univariate and multivariate Cox proportional hazards models were used. In the univariate Cox proportional hazards model, %FOtotal, ventilator, oliguria, SOFA score, days elapsed until CRRT after AKI, mean arterial pressure, congestive heart failure, chronic obstructive pulmonary disease (COPD), serum creatinine, platelet count, liver cirrhosis, prothrombin time (PT), and number of vasopressors were found to be *P* < 0.1. The variables with univariate *P* < 0.1 and sepsis status were entered into the multivariate Cox proportional hazard model. The sepsis status was intentionally included in the multivariate model because sepsis is known to be one of the most common causes of mortality in ICU patients.

We also performed the Kaplan-Meier survival analysis and Log rank test to compare survival among the 4 groups defined by %FOpreCRRT and %FOtotal, and in the various pre-defined subgroups. All tests were two-sided and *P* < 0.05 was considered significant. Statistical analyses were conducted using SPSS 21.0 (SPSS Inc., Chicago, IL).

## Results

Of the 341 patients in this study, 182 expired within 30 days after ICU admission. Baseline characteristics stratified by 30-day mortality are summarized in Table 1. In terms of demographics, there were no significant differences between survivors and non-survivors based on age, gender, and baseline body weight. Regarding coexisting disease, survivors had a higher prevalence of COPD, liver cirrhosis, and congestive heart failure. With regard to clinical features at ICU admission, non-survivors had lower levels of mean arterial pressure and platelet count. Non-survivors were also more likely to have a higher incidence of oliguria, along with elevated levels of serum creatinine and prothrombin time. According to severity of illness, non-survivors were more severely ill as indicated by higher SOFA scores, a greater number of vasopressors, and an increased frequency of ventilator care. However, there was no significant difference in the prevalence of sepsis between the 2 groups. Regarding fluid overload status, non-survivors had a higher incidence of total fluid overload (%FOtotal ≥ 10%). Finally, a greater number of days elapsed between AKI diagnosis and CRRT initiation in non-survivors compared to that in survivors.

**Table 1:** Baseline characteristics stratified by mortality at 30 days after ICU admission (n = 341).

Variables	Survivors (n = 159)	Non-survivors (n = 182)	P-value
**Demographics**			
Age (years)	59.4 ± 16.8	61.7 ± 16.2	0.19
Gender (male)	71.1%	67.0%	0.42
Baseline body weight (kg)	63.4 ± 9.3	61.7 ± 11.3	0.14
**Coexisting disease**			
Chronic kidney disease	20.1%	24.7%	0.31
Hypertension	43.4%	45.6%	0.68
Diabetes mellitus	23.3%	31.3%	0.10
COPD	4.4%	12.6%	0.007
Liver cirrhosis	9.4%	22.5%	0.001
Congestive heart failure	8.2%	22.0%	<0.001
**ICU location**			0.38
Medical ICU	76.7%	72.5%	
Surgical ICU	23.3%	27.5%	
**Findings at ICU admission**			
Mean arterial pressure (mmHg)	80.7 ± 19.0	72.1 ± 14.1	<0.001
Fever or hypothermia	18.9%	26.4%	0.10
Heart rate (beat/minute)	100.9 ± 43.5	103.6 ± 54.0	0.61
Oliguria (< 0.5 ml/kg per hour)	32.1%	47.8%	0.003
Serum creatinine (mg/dl)	4.4 ± 1.6	4.9 ± 2.3	0.02
Serum blood urea nitrogen (mg/dl)	81.2 ± 39.6	85.2 ± 49.3	0.41
Potassium (mEq/l)	4.8 ± 1.0	4.9 ± 1.0	0.28
Leukocyte count (1000/mm^2^)	14.2 ± 15.9	17.5 ± 25.9	0.17
Hemoglobin (g/dl)	10.5 ± 2.2	10.3 ± 2.4	0.46
Platelet count (1000/mm^2^)	153.2 ± 98.1	117.9 ± 85.2	<0.001
PT(INR)	1.4 ± 0.4	1.6 ± 0.6	0.009
pH	7.3 ± 1.3	7.3 ± 0.1	0.32
CRP (mg/dl)	13.7 ± 13.5	14.4 ± 11.7	0.63
**Severity of illness**			
Sepsis	58.5%	59.3%	0.87
SOFA score	10.0 ± 4.3	14.4 ± 4.0	<0.001
Number of vasopressors	0.9 ± 1.0	1.5 ± 1.0	0.001
Ventilator dependency	52.2%	79.1%	<0.001
**Fluid overload status**			
%FOtotal ≥ 10%^a^	25.8%	45.6%	0.001
**Days elapsed between CRRT initiation and AKI diagnosis**	2.2 ± 3.0	3.8 ± 2.9	<0.001

Values are expressed as the mean ± SD or percentage (%). AKI = acute kidney injury; COPD = chronic obstructive pulmonary disease; CRP = C-reactive protein; CRRT = continuous renal replacement therapy; ICU = intensive care unit; PT (INR) = prothrombin time (international normalized ratio); SOFA = Sequential Organ Failure Assessment

^a^%FOtotal ≥ 10% was defined as fluid overload ≥ 10% of baseline body weight from 3 days before CRRT initiation to ICU discharge.

**Fig 1:**
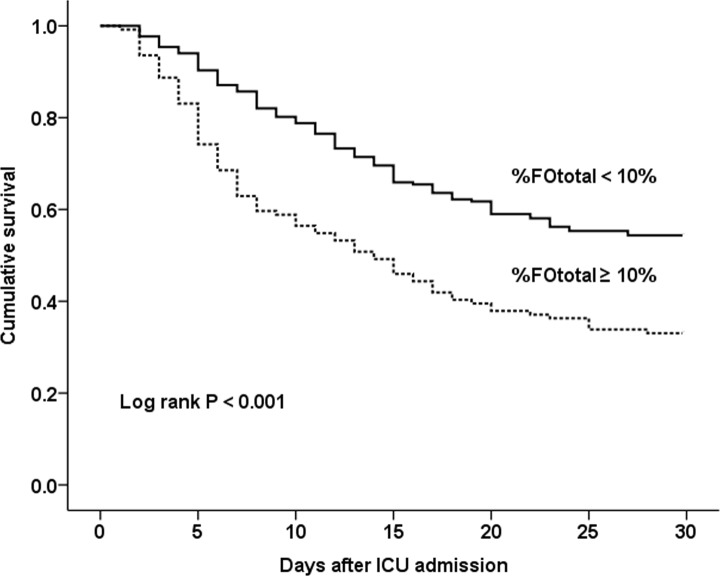
Kaplan-Meier survival estimates by fluid overload status (n = 341). There was a significant difference in survival among patients with %FOtotal ≥ 10% (n = 124) or < 10% (n = 217) (*P* < 0.001). %FOtotal = percentage of total fluid overload from baseline body weight from 3 days before CRRT initiation to ICU discharge. ICU = intensive care unit.

We assessed the association of total fluid overload with survival in all patients according to %FOtotal ≥ 10% (n = 124) or < 10% (n = 217). The patients with %FOtotal ≥ 10% showed a significant decrease in survival over 30 days after ICU admission compared to those with %FOtotal < 10% (30-day mortality: 66.9% vs. 45.6%, *P* < 0.001) (Fig 1). When the patients with %FOtotal ≥ 10% were further divided according to the percentage of fluid overload, the 30-day mortality was the highest in patients with %FOtotal ≥ 40% (n = 70, 81.8%), followed by 30% ≤ %FOtotal < 40% (n = 29, 78.6%), 20% ≤ %FOtotal < 30% (n = 14, 72.4%), and 10% ≤ %FOtotal < 20% (60.0%).

In univariate analyses, COPD (*P* = 0.006), liver cirrhosis (*P* = 0.003), congestive heart failure (*P* = 0.001), mean arterial pressure (*P* < 0.001), oliguria (*P* < 0.001), serum creatinine (*P* = 0.003), platelet count (*P* = 0.001), prothrombin time (*P* = 0.005), SOFA score (*P* < 0.001), number of vasopressors (*P* < 0.001), use of ventilator (*P* < 0.001), %FOtotal ≥ 10% (*P* < 0.001), and the time elapsed between AKI diagnosis and CRRT initiation (*P* < 0.001) were significant predictors of 30-day mortality (Table 2). In a multivariate Cox regression analysis for 30-day mortality after adjusting for sepsis status and variables that were significant in the univariate analysis, the presence of total fluid overload (%FOtotal ≥ 10%) was significantly associated with increased 30-day mortality (HR: 1.31, 95% CI: 1.13–2.06). Ventilator dependency (HR: 1.66, 95% CI: 1.08–2.54), oliguria (HR: 1.56, 95% CI: 1.13–2.14), SOFA score at ICU admission (HR: 1.15, 95% CI: 1.09–1.20), days elapsed between AKI diagnosis and CRRT initiation (HR: 1.03, 95% CI: 1.01–1.12), mean arterial pressure (HR: 0.99, 95% CI: 0.98–1.00), congestive heart failure (HR: 1.60, 95% CI: 1.09–2.37), and COPD (HR: 1.63, 95% CI: 1.01–2.66) were also significantly associated with increased 30-day mortality (Table 3). Sepsis, serum creatinine, platelet count, liver cirrhosis, PT (INR), and number of vasopressors were not associated with 30-day mortality.

**Table 2:** Univariate analysis for 30-day mortality after ICU admission in all subjects (n = 341).

Variables	HR	95% CI	P-value
**Demographics**			
Age (per year)	1.00	1.00–1.02	0.26
Male	0.94	0.69–1.23	0.68
Baseline body weight (per 1.0 kg increment)	0.99	0.97–1.00	0.10
**Coexisting disease**			
Chronic kidney disease	1.06	0.74–1.32	0.92
Hypertension	0.99	0.71–1.68	0.68
Diabetes mellitus	1.27	0.93–1.74	0.14
Chronic obstructive pulmonary disease	1.85	1.19–2.86	0.006
Liver cirrhosis	1.70	1.20–2.40	0.003
Congestive heart failure	1.83	1.28–2.59	0.001
**ICU location**			
Medical ICU (vs. surgical ICU)	1.05	0.76–1.45	0.79
**Findings at ICU admission**			
Mean arterial pressure (per 1.0 mmHg increment)	0.98	0.97–0.99	<0.001
Fever or hypothermia	1.27	0.92–1.77	0.15
Heart rate (per 1.0 beat/minute increment)	1.00	1.00–1.01	0.23
Oliguria (< 0.5 ml/kg per hour)	1.68	1.26–2.25	<0.001
Serum creatinine (per 1.0 mg/dl increment)	1.11	1.04–1.20	0.003
Serum blood urea nitrogen (per 1.0 mg/dl increment)	1.00	1.00–1.00	0.99
Potassium (per 1.0 mEq/l increment)	1.03	0.90–1.19	0.67
Leukocyte count (per 1000/mm^2^ increment)	1.00	1.00–1.00	0.43
Hemoglobin (per 1.0 g/dl increment)	0.99	0.93–1.06	0.80
Platelet count (per 1000/mm^2^ increment)	1.00	1.00–1.00	0.001
PT (INR) (per 1.0 increment)	1.42	1.11–1.80	0.005
pH (per 1.0 increment)	0.60	0.22–1.62	0.31
CRP (per 1.0 mg/dl increment)	1.00	0.99–1.02	0.41
**Severity of illness**			
Sepsis	1.16	0.86–1.56	0.33
SOFA score (per 1 point increment)	1.19	1.14–1.23	<0.001
Number of vasopressors (per 1 increment)	1.44	1.25–1.66	<0.001
Ventilator dependency	2.49	1.74–3.57	<0.001
**Fluid overload status**			
%FOtotal ≥ 10%^a^ (vs. < 10%)	1.90	1.42–2.55	<0.001
**Days elapsed between CRRT initiation and AKI diagnosis** (per 1 day increment)	1.09	1.04–1.13	<0.001

AKI = acute kidney injury; CI = confidential interval; CRP = C-reactive protein; CRRT = continuous renal replacement therapy; HR = hazard ratio; ICU = intensive care unit; PT (INR) = prothrombin time (international normalized ratio); SOFA = Sequential Organ Failure Assessment

^a^%FOtotal ≥ 10% was defined as fluid overload ≥ 10% of baseline body weight from 3 days before CRRT initiation to ICU discharge.

**Table 3:** Multivariate analysis for 30-day mortality after ICU admission in all subjects (n = 341).

Variables	Adjusted HR	95% CI	P-value
**%FOtotal ≥ 10%**^**a**^ **(vs. < 10%)**	1.31	1.13–2.06	0.01
**Ventilator dependency**	1.66	1.08–2.54	0.02
**Oliguria**	1.56	1.13–2.14	0.01
**SOFA score (per 1 point increment)**	1.15	1.09–1.20	<0.001
**Days elapsed between CRRT initiation and AKI diagnosis (per 1 day increment)**	1.03	1.01–1.12	0.04
**Mean arterial pressure (per 1.0 mmHg increment)**	0.99	0.98–1.00	0.04
**Congestive heart failure**	1.60	1.09–2.37	0.02
**Chronic obstructive pulmonary disease**	1.63	1.01–2.66	0.04
Sepsis	1.37	0.98–1.92	0.07
Serum creatinine (per 1.0 mg/dl increment)	1.07	0.99–1.16	0.66
Platelet count (per 1000/mm^2^ increment)	0.99	099–1.00	0.51
Liver cirrhosis	1.09	0.73–1.65	0.65
PT (INR) (per 1.0 increment)	1.00	0.75–1.33	0.99
Number of vasopressors (per 1 increment)	0.93	0.77–1.12	0.47

AKI = acute kidney injury; CI = confidential interval; CRRT = continuous renal replacement therapy; HR = hazard ratio; ICU = intensive care unit; PT (INR) = prothrombin time (international normalized ratio); SOFA = Sequential Organ Failure Assessment

^a^%FOtotal ≥ 10% was defined as fluid overload ≥ 10% of baseline body weight from 3 days before CRRT initiation to ICU discharge.

**Fig 2:**
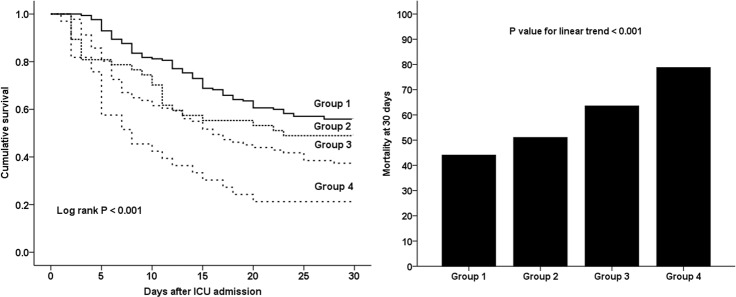
Kaplan-Meier survival estimate and 30-day mortality among the 4 groups categorized by %FOpreCRRT and %FOtotal. There was a significant difference in survival among the 4 groups (*P* < 0.001). 30-day mortality was the highest in Group 4, followed by Group 3, Group 2, and Group 1 (78.8% vs. 62.6% vs. 51.1% vs. 44.1%; *P* value for the linear trend < 0.001). Group 1: n = 170, %FOpreCRRT < 10% and %FOtotal < 10%; Group 2: n = 47, %FOpreCRRT ≥ 10% and %FOtotal < 10%; Group 3: n = 91, %FOpreCRRT < 10% and %FOtotal ≥ 10%; and Group 4: n = 33, %FOpreCRRT ≥ 10% and %FOtotal ≥ 10%. %FOpreCRRT = percentage of fluid overload from baseline body weight during 3 days prior to CRRT initiation. %FOtotal = percentage of total fluid overload from baseline body weight from 3 days before CRRT initiation to ICU discharge. ICU = intensive care unit.

We also compared survival among the 4 groups (Group 1: n = 170, %FOpreCRRT < 10% and %FOtotal < 10%; Group 2: n = 47, % FOpreCRRT ≥ 10% and %FOtotal < 10%; Group 3: n = 91, %FOpreCRRT < 10% and %FOtotal ≥ 10%; Group 4: n = 33, %FOpreCRRT ≥ 10% and %FOtotal ≥ 10%). A significant difference in survival among the 4 groups (*P* < 0.001) was shown, and 30-day mortality was the highest in Group 4, followed by Group 3, Group 2, and Group 1 (78.8% vs. 62.6% vs. 51.1% vs. 44.1%, respectively; *P* value for linear trend < 0.001) (Fig 2). We investigated the effect of starting CRRT within or after 72 hours from AKI diagnosis in each of the 4 groups. In Groups 1 and 2, the patients who received CRRT 72 hours after AKI diagnosis showed a higher 30-day mortality than those for whom CRRT was started within 72 hours of AKI diagnosis (Group 1: 64.4% vs. 36.8%, *P* = 0.009; Group 2: 72.7% vs. 32.0%, *P* = 0.007). In Groups 3 and 4, there were no significant differences among the patients who received CRRT within or after 72 hours of AKI diagnosis (Group 3: 72.4% vs. 58.1%, *P* = 0.33; Group 4: 78.6% vs. 72.1%, *P* = 0.99).

**Fig 3:**
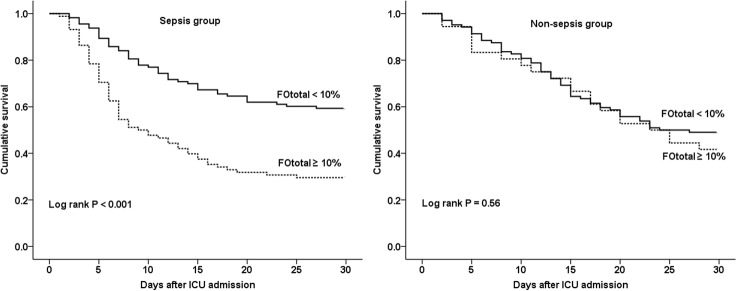
Kaplan-Meier survival estimate by %FOtotal ≥ 10% or < 10% in the sepsis group (n = 201) and the non-sepsis group (n = 140). In the sepsis group, there was a significant difference in survival among patients with %FOtotal ≥ 10% or < 10% (*P* < 0.001). However, in the non-sepsis group, there was no difference in survival (*P* = 0.56). %FOtotal = percentage of total fluid overload from baseline body weight from 3 days before CRRT initiation to ICU discharge. ICU = intensive care unit.

**Fig 4:**
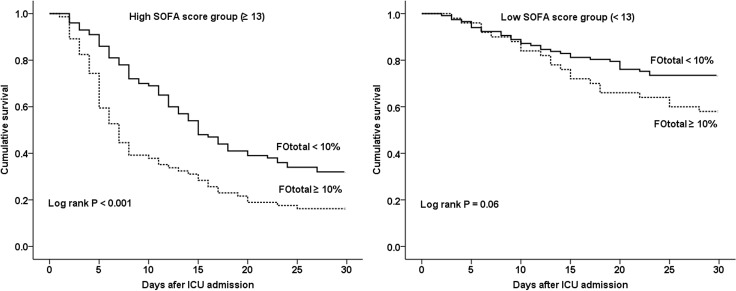
Kaplan-Meier survival estimate %FOtotal ≥ 10% or < 10% in the high SOFA score group (≥ 13, n = 174) and in the low SOFA score group (< 13, n = 167). In the high SOFA score group, there was a significant difference in survival among patients with %FOtotal ≥ 10% or < 10% (*P* < 0.001). However, in the low SOFA score group, there was no difference in survival (*P* = 0.06). %FOtotal = percentage of total fluid overload from baseline body weight from 3 days before CRRT initiation to ICU discharge. ICU = intensive care unit.

In the subgroup analyses, we investigated whether the adverse effect of fluid overload on survival could be applied to various subgroups. First, we classified all patients into either the sepsis group (n = 201) or non-sepsis group (n = 140). In the sepsis group, Kaplan-Meier survival estimate showed that patients with %FOtotal ≥ 10% had decreased survival over a period of 30 days compared to those with %FOtotal < 10% (*P* < 0.001). However, in the non-sepsis group, patients with %FOtotal ≥ 10% seemed to have decreased survival compared to those with %FO < 10%, but the difference was not statistically significant when the two survival curves were compared (*P* = 0.56) (Fig 3). Second, we divided all patients into a high SOFA score group (13 ≥, n = 174) and a low SOFA score group (< 13, n = 167) according to severity of illness. In the high SOFA score group, there was a statistically significant difference in survival among patients with %FOtotal ≥ 10% or %FOtotal < 10% (*P* < 0.001); however, this difference was not observed in the low SOFA score group (*P* = 0.06) (Fig 4).

## Discussion

Fluid therapy is an essential component of caring for patients who are critically ill in terms of the maintenance of hemodynamic stability. Early and timely fluid administration targeted to appropriate physiologic end-points has been considered vital in resuscitating patients with septic shock and severe sepsis since the results of the landmark Early Goal-Directed therapy (EGDT) study were published [14]. However, beyond the threshold needed for acute resuscitation, fluid overload may actually cause harm. Indeed, fluid overload has been reported to be associated with increased mortality in critically ill patients without AKI, including septic shock and acute lung injury [15–18]. Among critically ill patients with AKI, several observational studies have reported an association between fluid overload and increased mortality. This was initially demonstrated in a few studies involving critically ill pediatric patients with AKI [8–10]. Recently, this association was confirmed by two notable studies investigating adult patients with AKI [11, 12]. The Sepsis Occurrence in Acutely Ill Patients (SOAP) study investigated the influence of fluid balance on the survival of critically ill patients with AKI [11]. Among 3,147 patients, 1,120 developed AKI and the 60-day mortality was higher in the patients with AKI (36%) compared with those without AKI (15%). In the patients with AKI, a positive fluid balance (1 liter/24 hours) was associated with an approximately 20% increase in 60-day mortality risk. In the PICARD study, the association between fluid overload and mortality in critically ill patients with AKI was more clearly established [12]. Of the 618 critically ill patients with AKI, those with fluid overload, defined as a greater than 10% increase in body weight relative to baseline, showed higher mortality at 30 days (37% vs. 25%), 60 days (46% vs. 32%), and at hospital discharge (48% vs. 35%) than those without fluid overload. Among dialyzed patients, survivors had a lower percentage of fluid accumulation at dialysis initiation compared with non-survivors (8.8% vs. 14.2%). Patients with fluid overload at dialysis initiation were approximately two times more likely to die compared to patients without fluid overload. Furthermore, this study showed that the proportion of dialysis days with fluid overload was associated with increased mortality, suggesting a cumulative effect of fluid overload on mortality.

Our study supports the adverse effect of fluid overload on survival, which is consistent with previous reports. Patients with AKI who had fluid overload (> 10% from baseline body weight) from 3 days before CRRT initiation to ICU discharge had significantly decreased survival compared with those who did not have fluid overload. The patients with fluid overload were 1.31 times more likely to die within 30 days after ICU admission than those without fluid overload. Moreover, fluid removal by CRRT was associated with a reduction in mortality. In the subgroup analysis, 30-day mortality was the highest in Group 4 (78.8%, %FOpreCRRT ≥ 10% and %FOtotal ≥ 10%), followed by Group 3 (62.6%, %FOpreCRRT < 10% and %FOtotal ≥ 10%), Group 2 (51.1%, FOpreCRRT ≥ 10% and %FOtotal < 10%), and Group 1 (44.1%, %FOpreCRRT < 10% and %FOtotal < 10%). This finding not only showed that attaining a total fluid overload < 10% during CRRT was associated with decreased mortality, but it also demonstrated the importance of achieving a total fluid overload of < 10% via fluid removal by CRRT when the fluid overload existed prior to CRRT initiation. Currently available therapies for fluid overload include the use of diuretics and dialysis. Previous studies have shown that diuretics did not have a significant effect on mortality [19, 20] and might be associated with worse survival in patients with AKI [6]. Moreover, the use of diuretics is often limited by diuretic resistance. Thus, clinicians have no choice but to start dialysis in patients with AKI and fluid overload who are unresponsive to diuretics. During intermittent hemodialysis (IHD), patients who are critically ill often experience intradialytic hypotension, and further ischemic injury to the kidney can occur. CRRT may be more advantageous due to its superior maintenance of hemodynamic stability. In the PICARD study, it was shown that patients treated with CRRT were more likely to experience reduced fluid overload when compared with those treated with IHD, and that mortality was lower when fluid overload was corrected by dialysis therapy [12]. However, we think the results of our study need to be interpreted cautiously with regard to whether the survival benefit of fluid removal via CRRT is due to its direct therapeutic effect or to the fact that patients with less severe clinical conditions can tolerate ultrafiltration.

One of the significant prognostic factors of mortality in this study was the amount of time elapsed until CRRT initiation following AKI diagnosis. The timing of CRRT initiation remains controversial due to the difficulty in defining early vs. late initiation. Several retrospective studies have suggested the early initiation of CRRT in critically ill patients with AKI [21–23], while one prospective randomized study found no difference in survival between early and late CRRT initiation [24]. Our study showed that the number of days elapsed between AKI diagnosis and CRRT initiation was significantly associated with increased mortality. Although this study has some limitations, including its retrospective design, the results support the idea that the early initiation of CRRT in critically ill patients with AKI is important in improving the prognosis.

The main finding of this study is that the detrimental effect of fluid overload on survival is more evident in patients with sepsis or more severe illness than in those without sepsis or with less severe illness. In patients with sepsis or a high SOFA score, there was a significant difference in survival among the patients with and without fluid overload. In patients without sepsis or with a low SOFA score, there was no difference in survival among the patients irrespective of fluid overload. The reason for this remains unclear, and further large, prospective, controlled studies are needed to confirm these results. However, there are some points to be considered with regard to septic AKI. Traditionally, septic AKI has been considered an ischemic form of AKI, and increasing renal perfusion by means of fluid resuscitation had been considered the cornerstone of treatment. However, recent studies have suggested that septic AKI and non-septic AKI might have distinct pathophysiologies, and that the rationale for aggressive fluid administration in septic AKI beyond the initial resuscitation point should be questioned [25–28]. Thus, the different effects of fluid overload on survival might result from the different pathophysiologies of septic and non-septic AKI.

There are several limitations in our study. First, owing to its retrospective design, it is not possible to discern whether fluid overload is a marker of more severe disease or a casual contributor to mortality in our study subjects. However, recent reports have suggested that fluid overload itself could lead to increased mortality due to its direct toxic effect on organ function [29–31], and we contend that fluid overload might have a direct effect on mortality in critically ill patients with AKI, though this needs to be verified in a well-designed, randomized controlled study. Second, this study only included data about fluid balance obtained from 3 days before CRRT initiation to ICU discharge due to a lack of clinical information. A more prolonged observation period such as from the date of AKI diagnosis to hospital discharge might more clearly reveal the association between fluid overload and survival. Third, the subjects in our study were a specific subset of patients with AKI, namely those who were critically ill and received CRRT. Thus, our results were confined to this specific cohort and might not be extrapolated to other populations of patients with AKI.

In conclusion, our study demonstrated that fluid overload was independently associated with mortality, and that fluid removal by CRRT appears to reduce mortality in critically ill patients with AKI. This study also showed that the adverse effect of fluid overload on survival was more evident in patients with sepsis or more severe illness, suggesting that a more rigorous effort to reduce fluid overload is necessary in those patients, which might not apply to patients without sepsis or with less severe illness.
